# ARMC2 loss impairs cilia structure and leads to primary ciliary dyskinesia symptoms in mouse organs

**DOI:** 10.3389/fcell.2026.1695239

**Published:** 2026-05-26

**Authors:** Elsa Giordani, Solène Houdeline, Magali Court, Sylvie Gory-Fauré, Jean-Christophe Deloulme, Anne-Pascale Bouin, Fabrice Senger, Charles Coutton, Edgar Del Llano, Anne-Laure Barbotin, Angèle Boursier, Geneviève Chevalier, Anne Bertrand, Christophe Bosc, Jessica Escoffier, Pierre F. Ray, Guillaume Martinez, Christophe Arnoult, Corinne Loeuillet

**Affiliations:** 1 Institute for Advanced Biosciences, University Grenoble Alpes, INSERM U1209, CNRS UMR 5309, Team Genetics Epigenetics and Therapies of Infertility, Grenoble, France; 2 Grenoble Institut Neurosciences (GIN), University Grenoble Alpes, Inserm, U1216, Grenoble, France; 3 Institute for Advanced Biosciences, University Grenoble Alpes, INSERM U1209, CNRS UMR 5309, Team Dynamique de l’adhésion cellulaire et différentiation, Grenoble, France; 4 CHU Grenoble Alpes, UM GI-DPI, Grenoble, France; 5 Laboratory of Development and Plasticity of the Postnatal Brain, University Lille, Inserm, CHU Lille, Lille Neuroscience & Cognition, UMR-S1172, FHU 1000 days for health, Lille, France; 6 Laboratoire de Biologie de la Reproduction-Spermiologie-CECOS, CHU Lille, Lille, France

**Keywords:** ARMC2, cilia, MMAF, PCD, sperm flagella

## Abstract

In humans and mice, deficiency in ARMC2 causes Multiple Morphological Abnormalities of the Flagellum (MMAF), a condition defined by absent or aberrant sperm flagella with a disorganized axoneme. Affected men are infertile but with no other obvious signs characteristics/typical for primary cilia dyskinesia (PCD). Given the similarity between cilia and flagella axonemes we investigated a possible role of ARMC2 in cilia functioning. In *Armc2*-deficient mice, the length of cilia was reduced in trachea and oviduct. Ciliary beating was also affected, leading to tracheal mucus accumulation and female mutants had fewer pups, likely due to impaired oviductal transport. Additional PCD manifestations included enlarged brain ventricles and occasional severe hydrocephalus, and situs ambiguus were also observed. Altogether, these findings suggest that mutations of *ARMC2* might be also a molecular cause of human PCD.

## Introduction

Cilia are ubiquitous microtubule-based organelles that project from the surface of most eukaryotic cells and are involved in motility or signal transduction. Cilia are classified according to their number per cell and their motility properties (Bernabé-Rubio and Alonso, 2017). Primary cilia, a most common sensory cilia, are immotile and assembled as a single structure per cell by diverse cell types including epithelial cells, hair cells, and photoreceptors. Nodal cilia, although also present as a single organelle per cell, are exceptional motile cilia in the embryonic node, whose rotational beating generates a leftward fluid flow that establishes left-right organs’ patterning. Defects in this process can lead to laterality disorders, including heterotaxy (Ibanez-Tallon, 2003).

Motile cilia are formed/assembled either in large numbers on multiciliated epithelia (epithelium lining the airway, oviduct/fallopian tube, efferent ductules, and ependymal cells lining the brain ventricle), where they propel biological fluids or as a single structure. For instance, spermatozoa and certain unicellular organisms, such as *Chlamydomonas,* possess a flagellum, a homologous organelle to cilia. Both cilia and flagella share the same core architecture (the axoneme), and are derived from basal body (Ibanez-Tallon, 2003). Consistent with their broad distribution and specialized functions, ciliary dysfunction causes a wide spectrum of tissue-specific pathologies. In the central nervous system, impaired ependymal multicilia compromise cerebrospinal fluid (CSF) flow and clearance (Faubel et al., 2016). In the upper respiratory tract, defective motile cilia reduce mucociliary clearance, leading to mucus accumulation and recurrent infections (Ibanez-Tallon, 2003). In females, ciliary defects can cause subfertility or infertility by impairing oocyte pickup at the infundibulum and reducing the efficiency of gamete and embryo transport within the fallopian tube (Talbot et al., 1999; Lyons et al., 2006; Harwalkar and Yamanaka, 2021). In males, defects in the cilia of the efferent ductules within the caput epididymis can disrupt sperm transport and maturation (Yuan et al., 2019; Wloga et al., 2024) and flagellar dysfunction can compromise sperm motility (Demott and Suarez, 1992).

The immotile primary cilia harbor an axonemal 9 + 0 architecture composed of nine microtubules (MT) doublets forming a wheel. In the motile cilia and flagella, this axonemal structural core surrounds a central pair apparatus formed by two singlet microtubules. This 9 + 2 architecture is characteristic of most motile cilia and flagella, which therefore share key structural and functional features (Bernabé-Rubio and Alonso, 2017; Newman et al., 2023). Motility is driven by the orchestrated activity of the conserved axonemal components including central apparatus, radial spokes, N-DRC, and dynein arms.

Flagellar and ciliary biogenesis begins with the migration of centrioles toward the cell surface, where they mature into basal bodies and initiate ciliogenesis. From the ciliary base, axoneme elongation and ciliary membrane biogenesis rely on two complementary modes of delivery: small soluble molecules can enter by passive diffusion (Kee et al., 2012) whereas larger and 50 kDa proteins and precomplexes are transported by the intraflagellar transport (IFT) machinery. More generally, IFT trains shuttle a wide range of cargos required for axoneme assembly, function, and maintenance (Nakayama and Katoh, 2020). Ciliogenesis is thus a highly coordinated process that relies heavily on a large number of proteins, and defects in its components can lead to a broad spectrum of ciliopathies (Fliegauf et al., 2007).

Ciliopathies are complex genetically heterogeneous disorders caused by defects in at least 247 genes that affect primary cilia, motile cilia, or both (Wallmeier et al., 2020; Mill et al., 2023). Most of the identified ciliopathy genes encode proteins that localize along the cilium-basal body axis and are implicated in so-called first-order ciliopathies, whereas genes mutated in second-order ciliopathies encode proteins situated elsewhere in the cell, exerting indirect effects on ciliary structure or function. Primary cilia dyskinesia (PCD) is characterized by recurrent respiratory tract infections, infertility/subfertility, and in about 50% of patients - by laterality defects (Kartagener’s syndrome) (Raidt et al., 2023). Mutations in over 50 genes encoding diverse ciliary components have been identified as causal in PCD (Raidt et al., 2022). These mutations can affect the outer dynein arms (ODA) complex (*DNAI1, DNAI2, DNAH5, DNAH9, DNAL1), ODA associated proteins (CCDC103, NM8),* ODA docking complexes (*CCDC114, ARMC4, CCDC151, TTC25, CLXN, DAW1*), the inner dynein arms (IDA) (DNAH7), assembly factors *(LRRC50, KTU, C19ORF51, DYX1C1, HEATR2, ZMYND10, LRRC6, SPAG1, CFAP298, CFAP300, TTC12), central pair components (HYDIN, SPEF2, CFAP74, CFAP221, STK36), radial spokes (RS) head components (RSPH1, RSPH4A,* RSPH9) RS stalk *components* (RSPH3, RSPH23, DNAJB13), the nexin-dynein regulatory complex (N-DRC) (CCDC164, CCDC65, GAS8), and the axonemal ruler *(CCDC39*, *CCDC40)* (Raidt et al., 2023). Recently, infertile men have been described with severe anomalies of the sperm flagella without other signs of PCD, a condition described as multiple morphological anomalies of the sperm flagella (MMAF). At least 50 different genes have been associated with MMAF so far, many of them coding for axonemal and peri-axonemal proteins or factors involved in flagella formation (Cavarocchi et al., 2025).

We previously identified *ARMC2* as a MMAF gene in both humans and mice (Coutton et al., 2019). In the mutant mouse line, a one-nucleotide duplication in exon 4 (c.403dupT), introduces a translational frameshift leading to production of abnormal transcripts with a premature stop codon 12 nucleotides after the first modified codon at position 135 (GenBank: NM_001034858.3 [c.403dupT]; NCBI: NP_001030030.2 [p.Tyr135LeufsTer12]). Homozygous male mice are infertile, and their sperm present a characteristic MMAF phenotype having absent, curved, or shorter flagella, and severely impaired motility. At the ultrastructural level, the sperm central pair complex (CPC) was absent from the flagellum, whereas other structures were not affected. This CPC structural defect was also reported in patients carrying an *ARMC2* mutation (Khan et al., 2021; Wang et al., 2022).

In *Chlamydomonas reinhardtii*, ARMC2 is involved in IFT transport of RS components (Lechtreck et al., 2022). Specifically, ARMC2 act as an adaptor for the IFT of RS components, supported by several lines of evidence: in pf27 and *Armc2* mutants, RS were confined to the proximal region of the flagellum, consistent with defective RS transport and/or assembly. Accordingly, no intraflagellar transport (IFT) of tagged RSs were detected in *Armc2* mutants. Moreover, tagged ARMC2 co-migrates with the essential radial spoke protein RSP3 on anterograde IFT trains, supporting the model that ARMC2 functions as a transport adaptor for RS precursors (Lechtreck et al., 2022).

Multiple lines of evidence indicate that defects in, or perturbations of the IFT machinery can induce a PCD phenotype. Mutations in IFT74 (IFT-B complex) cause a combined motile and primary ciliopathy that includes a PCD-like airway disease (Fassad et al., 2023). LRRC56, an IFT-associated cargo protein, has been linked to impaired mucociliary clearance and laterality defects (Bonnefoy et al., 2024). More recently, in *Trypanosoma brucei*, LRRC56 was shown to ride on IFT trains and to be required for the docking and assembly of distal ODA (Bonnefoy et al., 2024). Consistent with the tight coupling between the dynein-arm pathways and IFT, deletion of *ZMYND10* in *Paramecium tetraurelia* leads to an abnormal distribution of IFT43 and severe defects in ciliary structure and function of cilia (Shi et al., 2021). Collectively, these data support the idea that disruption of core IFT components, or dynein-arm assembly factors whose trafficking depends on IFT, can manifest as a PCD-like respiratory phenotype.

Interestingly, in addition to its high expression in testis, databases indicate that ARMC2 is also expressed in different ciliated tissues including the respiratory system and cerebral cortex (Uhlén et al., 2015). Interestingly, a patient carrying bi-allelic *ARMC2* variants was recently reported to present pulmonary dysfunction in addition to flagellar anomalies, (Baoyan et al., 2025). Here, we investigate the role of ARMC2 across distinct ciliated tissues and test whether its loss can lead to PCD-like phenotype in addition to MMAF. Overall, our findings identify ARMC2 as a contributor to ciliogenesis and show that its absence compromises cilia-dependent functions in the reproductive, neuronal and respiratory systems.

## Materials and methods

### Mice

Animals were housed and bred at « Plateforme de Haute Technologie Animale (PHTA)» UGA core facility hTAG, Inserm US46, CNRS UAR2019 (La Tronche, France), EU0197, Agreement D38-516 10,006, under specific pathogen–free conditions, in a temperature-controlled environment with a 12 h light/dark cycle and *ad libitum* access to food and water. Animal housing and procedures complied with European Communities Council Directive 2010/63/EU and according to recommendations for health monitoring from the Federation of European Laboratory Animal Science Associations. All animals’ protocols were reviewed by the local ethics committee “Comité d’Ethique pour l’Expérimentation Animale no.#12, Cometh-Grenoble” and approved by the French Ministry of Research (ministry agreement number #7128 UHTA-U1209-CA). The *Armc2* knockout (*Armc2*-KO) mouse line used in this study was previously described (Coutton et al., 2019).

The colony was maintained by mating heterozygous animals, and heterozygous and KO mice were identified by genotyping. For all experiments, mice were sacrificed by cervical dislocation.

### Genotyping

Genomic DNA was extracted from ear punches by overnight digestion at 55 °C in Direct PCR lysis buffer (Euromedex, Souffelweyersheim, FR) supplemented with 1 mg/mL proteinase K (Thermo Fisher Scientific, Waltham, Massachusetts, USA), followed by enzyme inactivation for 1 h at 85 °C. PCR was performed as follow: 2 µL of undiluted DNA was mixed with 0.4 µM primers against *Armc2* gene (Genscript, Piscataway, NJ, USA, sequences are listed in Table 1), Coral Load PCR Buffer 1X (Qiagen, Hilden, DE), 0.8 mM dNTP mix (Thermo Fisher Scientific) and 0.625 units of Taq DNA Polymerase (Qiagen) in a final volume of 25 µL. Cycling conditions were 94 °C for 15 min (94 °C 30 s, 59 °C 30 s, 72 °C 1 min) x 32 followed by a final elongation (72 °C 10 min). PCR products were then visualized by electrophoresis (BioRad, Hercules, California, USA) on a 1% agarose gel with Gelgreen 1,8X (Interchim, Montluçon, FR).

**TABLE 1:** Primer sequences used for mouse genotyping and *Armc2* expression.

Genotyping	Primer	Primer sequences (5′-3′)	Concentration (µM)
Genotyping			
*Armc2_WT*	Forward	GGCCCGAGCACGCTTCTA	0.4
	Reverse	TTC​ATG​TAA​GAA​CTA​TCC​AGG​ACC​A	0.4
*Armc2_KO*	Forward	TGGGACGCAGCCCTGTAA	0.4
	Reverse	AAC​CCA​AAG​CTC​CAG​CAT​CTC	0.4
Expression			
*Actb*	Forward	CTT​CTT​TGC​AGC​TCC​TTC​GTT​GC	0.25
	Reverse	GCT​GGT​CGT​CGA​CAA​CGG​CT	0.25
*Armc2*	Forward	ACT​CGA​AAA​GCT​GGA​TTC​CT	0.50
	Reverse	CCA​TTC​CTT​CTC​GCT​GTA​GA	0.50

### RNA extraction and quantitative PCR

Up to 30 mg of brain, trachea, lung, liver, spleen, kidney, oviduct and testis from mature wild-type (WT) mice were snapfrozen in liquid nitrogen and grinded with pestle and mortar. RNAs were extracted following the manufacturer’s recommendations using the NucleoSpin RNA Plus kit (Macherey Nagel, Düren, Nordrhein-Westfalen, DE). After final elution, RNAs concentrations were determined by using the Qubit RNA assay kit (Life Technologies, Carlsbad, Californie, USA) and the RNAs were then stored at −80 °C until use. Eight hundred ng of each tissue RNA was reverse transcribed using the iScript cDNA synthesis kit (BioRad) as recommended. Gene expression was assessed by qPCR (1 μL of undiluted cDNA in a final volume of 20 μL with the appropriate quantity of primers, see Table 1) using the SsoAdvanced Universal SYBR Green Supermix (BioRad). The qPCR program used was 94 °C 15 min (94 °C 30 s, 58 °C 30 s, 72 °C 30 s) x 40 followed by a melt curve analysis (58 °C 0.05 s, 58 °C–95 °C 0.5 °C increment 2–5 s/step). Relative expression levels were calculated using the 2^−ΔΔCT^ method.

### Immunofluorescence on tissue explant

Mice tracheal explants were fixed in 4% paraformaldehyde (PFA, Biovalley) in 1X PBS (pH 7.4) for 45 min at 4 °C. After fixation, samples were washed three times for 5 min each in 1X PBS at room temperature to remove excess fixative. Explants were permeabilized in 0.5% Triton X-100 in 1X PBS for 10 min at room temperature with gentle agitation. Following permeabilization, samples were rinsed three times in 1X PBS. Non-specific binding sites were blocked by incubating the explants in 5% normal goat serum (Sigma Aldrich) in 1X PBS for 1.5 h at room temperature. Explants were incubated overnight at 4 °C with primary antibodies (Table 2) diluted in 1% NGS in 1X PBS. After primary antibody incubation, explants were washed three times for 10 min each in 1X PBS at room temperature. Explants were then incubated for 1.5 h at room temperature with secondary antibodies (diluted 1:800 in 1% NGS in 1X PBS). Following secondary antibody incubation, explants were washed three times for 10 min each in 1X PBS. Explants were placed on Cellview slides (Dutscher), gently flattened with a coverslip and a metal ring to ensure even compression. Images were acquired using a Zeiss Axio Imager seven microscope with a ×100 oil immersion objective.

**TABLE 2:** Primary and secondary antibodies used for immunofluorescence.

Primary antibodies			
Target protein	Host species	References	Dilution
ARMC2	Rabbit	HPA053696, Atlas antibodies	1/50
Acetylated tubulin	Mouse	T7451, Sigma-Aldrich	1/1000
Gamma tubulin	Mouse	66320-1-Ig, proteintech	1/500
RSPH1	Rabbit	HPA017382, Atlas antibodies	1/100
RSPH4a	Rabbit	HPA03196, Atlas antibodies	1/100

Secondary antibodies			
Target species	Fluorophore	References	Dilution
Goat anti-rabbit	Alexa Fluor 568	A-11036	1/800
Goat anti-mouse	Alexa Fluor 488	A-11001	1/800
Goat anti-rabbit	Alexa fluor 647	A-21244	1/800

### In silico analysis

The AlphaFold-Multimer complex was predicted from the protein sequences of *Armc2*, *Rsph1 and Rsph4* using ColabFold with default parameters. Model confidence was assessed using the output metrics, including iPTM and pTM. Uncertainty in the relative inter-chain geometry was evaluated using the PAE (Predicted Aligned Error) matrix.

### Histology

Trachea and oviduct were recovered from *Armc2*-KO and *Armc2*-WT mice and fixed in paraformaldehyde (PFA) 4% overnight (Electron Microscopy Sciences, Hatfield, PA, USA). Tissues were then washed 3 times in PBS 1X and dehydrated in successive baths of 70%, 90%, 100% ethanol (Honeywell, Charlotte, North Carolina, USA) for 1 h each followed by bath in xylene for 1 h (Carl Roth) before being embedded in paraffin (Leica Biosystems, Nussloch, DE). Sections of 5 µm were cut with a microtome (Leica Biosystems) and placed onto Epredia™ Polysine Adhesive slides (Fischer scientific). The paraffin on slides was dissolved using xylene, tissues were rehydrated in ethanol (100%, 95%, 70%) before being stained for 15 s with hematoxylin solution (Sigma Aldrich) and counterstained for 2 min with Eosin Y solution (Sigma Aldrich). Tissue morphologies were observed after slide mounting with Eukitt (Dutscher, Bernolsheim, FR) with an Axio Observer 7, Zeiss microscope, oil objective ×63 (Zeiss, Oberkochen, DE).

For the brain, after cervical dislocation, mice were transcardially perfused with PFA 4%. The brains were removed from the skull, fixed overnight in PFA 4% at 4 °C, placed in ascending concentrations of sucrose (15% and 30% in PBS 1X) until they sank, frozen in −40 °C isopentane and stored at −80 °C. Brains were sectioned and floating coronal sections (60 μm thick) were stained with cresyl violet.

### Brain preparation for *ex vivo* T2 weighted MRI acquisitions

T2-weighted magnetic resonance imaging (MRI) was performed *ex vivo* on mouse brains. Following cervical dislocation, mice were decapitated and the heads were immersion-fixed in PFA 4% for 2 weeks. After removing surrounding skin and muscles, skulls containing intact brains were further fixed for 7 days and then transferred to a Fomblin (FenS chemicals, Amundsenweg, Amsterdam, NL) bath prior to imaging.

### Magnetic resonance imaging (MRI)

MRI was performed at 9.4T Bruker Biospec Avance III (Bruker, Billerica, Massachusetts, USA) system with a volume transmit/surface receive coil combination at the IRMaGe platform (Grenoble institute of Neuroscience). The skull was placed in cradle, and T2-weighted spin-echo images were acquired (TR/TE = 2500/33 m, field of view = 22 × 22 mm^2^, slice thickness 0.7 mm, 17 slices) covering the entire brain. The total scan duration was 20 min per skull.

### Scanning electron microscopy

Tracheal and oviductal explants from *Armc2*-WT and *Armc2*-KO mice were recovered in PBS 1X and dissected as followed. Tissues surrounding the trachea were removed and a longitudinal opening was made in the muscular side with a micro-scissor to expose the lumen of the tube. For the oviduct, the part proximal to the ovary known as the infundibulum was separated from the rest of the oviduct under binocular loupe thanks to a scalpel blade and an 18G needle. Both tissue explants were fixed in PBS 1X, glutaraldehyde 2.5% (Sigma Aldrich), PFA 2% (Electron Microscopy Sciences) and cacodylate 0.1M (Sigma Aldrich) pH 7.2 for 2h at RT. After three washes in 0.1 M cacodylate buffer, the tissues were post-fixed in osmic acid 2% (Sigma Aldrich), 0.1 M cacodylate for 1 h on ice. The samples were then washed, dehydrated with increasing baths of ethanol (30%, 60%, 90% and 100%, Honeywell), desiccated in hexamethyldisilazane (Carl Roth) and air-dried overnight. Finally, explants were metalized with 3 nm gold palladium particles thanks to Gatan 682 PECS before being imaged at 3 kV using In-Lens detectors of a scanning electron microscope Ultra 55, Zeiss at the C.M.T.C. (Consortium des Moyens Technologiques Communs (Material 913 characterization platform)), Grenoble INP. The measure of the cilia length of trachea and oviduct was determined by using the freehand line in FIJI software.

### Transmission electron microscopy

*Armc2*-KO and *Armc2*-WT animals were euthanized by cervical dislocation and intracardially perfused with glutaraldehyde 1.7% (Sigma Aldrich) in PBS 1X. The trachea and the oviduct’s infundibulum were recovered and post-fixed for 1 h on ice in 1% tetroxide osmium in PBS. After three washes, samples were stained in uranyl acetate 0.5% pH four in distilled H_2_O on ice (Taab, Berkshire, GB). The sample was then dehydrated in graded ethanol series: 30%, 60%, 90% and 100% before embedding with the resin Epoxy Embedding Kit (Sigma Aldrich). After polymerization of the resin for 2 days at 60 °C, ultrathin sections of 50 nm were cut with a diamond knife (Ultra 45°, Diatome) using an ultramicrotome (Reichert ultracut S, Leica Biosystems). Samples were put into gilder grids (150 mesh Cu/Pd, Electron Microscopy Sciences) and stained with uranyl acetate 5%, pH 4.0 in distilled H_2_O, followed by a coloration with 4 mg/mL of lead citrate (AGAR scientific, Sheffield, GB). Samples were observed with a transmission electron microscope at 80 kV (Jeol 1200 EX, Croisy-sur-Seine, FR) and images were acquired with a digital camera (Veleta, Olympus, Rungis, FR) and iTEM software.

### High speed videomicroscopy

For oviductal cilia beat frequency determination, *Armc2*-KO and *Armc2*-WT females were synchronized at the estrus stage by intraperitoneal injection (i.p.) of PMSG 5U (MSD santé animale) followed 48 h later by i.p. of hCG 5U (MSD santé animale, Pluteaux, FR). Twelve hours after hCG injection, the oviduct’s infundibulum was recovered and placed in M199 media (Sigma) supplemented with 10% of FBS (Life technologies), 1% of penicillin/streptomycin (Sigma Aldrich) at 37 °C (M199/FBS). The estrus stage was checked by the presence of eggs in the ampulla and the infundibulum was placed into a Bioptech Delta T culture dish (AutoMate Scientific, Berkeley, California, USA) in 100 µL of M199/FBS.

Tracheal dissection was based on the Francis *et al* work (Francis and Lo, 2013). The trachea was recovered in L15 (Sigma Aldrich) medium supplemented with 10% FBS and 1% penicillin/streptomycin (L15/FBS) supplemented with 20 mM of HEPES (Sigma Aldrich) and 0.5% of methylcellulose (M0512, Sigma Aldrich) at 37 °C. The tracheal tissue was placed in a Bioptech dish, the face harboring cilia facing the dish bottom, in 100 µL of L15/FBS.

For both tissues, 10 s videos were acquired at 37 °C using Bioptechs Delta T Culture Dish Controller (Bioptechs, Paris, FR) at 400 frames per s (fps) using a high-speed video camera (Camera C14440-20UP ORCA FUSION, Hamamatsu, Nikon, Minato-ku, Tokyo, JP), a ×100 oil objective and an Axio Observer 7 Zeiss microscope. CBF (Ciliary Beat Frequency) was determined using FIJI software. Briefly, a line was set parallel to the cell membrane and a kymograph of cilia motility was generated using the FIJI Multi kymograph plugin from the movie. Then, by applying the fast Fourier transform (FFT) algorithm, the dominant frequency (corresponding to half the distance between the highest peak in the FFT plot) was identified. Finally, the CBF was determined using the following equation:

$$CBF\;\left(Hz\right)=\text{fps }x\;\frac{\left(\frac{\text{distance}\;\text{between}\;\text{the}\;\text{highest}\;\text{peak}}{2}\right)}{4096}$$

To determine flow velocity, the tracheal explant was placed in the L15/FBS/0.5% methylcellulose and the infundibulum was placed in M199 medium. For both, pre-washed 0.53 µm fluorescent beads (Polyscience, Warrington, PA, USA) at a concentration of 5.46 × 10^9^ particles/ml were added. The tissues were maintained at 37 °C in the supplemented medium. The motion of fluorescent microspheres was analyzed using the TrackMate plugin in ImageJ, which is specifically designed for tracking and analyzing particles in microscopy images and time-lapse videos. Subsequently, a custom macro in ImageJ was used to determine the velocity of each particle (segmented into 15-frame intervals) and measure the distance between each particle and the user-defined ciliary line.

### Fertility testing

B6D2 WT male mice were mated with *Armc2*-KO or *Armc2*-WT female mice over a period of 4 months. The number of litters, the number of pups per litter and the time between litters were recorded.

### Embryo development assay

The rate of embryonic development was analyzed as follow. *Armc2*-WT or *Armc2*-KO mice were placed overnight with WT C57BL6/J males (ratio 1:1). The following day, mating was confirmed by the presence of a plug. Female mated mice were sacrificed by cervical dislocation, and the reproductive tract was recovered in pre-warmed 37 °C M2 (Sigma Aldrich). The cumulus-oocyte complexes were removed from ampulla and treated for 5 min with 10 μg/mL of hyaluronidase (Sigma Aldrich) to remove the cumulus cells. The eggs were then transferred in KSOM solution (Table 3) supplemented with 1 mg/mL BSA (Sigma Aldrich); non-essential amino acid 1X (Thermo Fisher Life Technologies); essential amino acid 1X (Thermo Fisher Life Technologies); 1% penicillin/streptomycin (Sigma Aldrich) and 1.71 mM CaCl_2_ (Sigma Aldrich) and covered with mineral oil (Sigma Aldrich). Fertilized eggs were incubated for 4 days at 37 °C, CO_2_ 5% under a humidified atmosphere and embryonic development was monitored daily until the blastocyst stage.

**TABLE 3:** KSOM media composition used for embryo development assay.

KSOM media		
Product	Concentration (mM)	Supplier
NaCl	95	Euromedex
KCl	2.55	VWR, Fontenay-sous-Bois, FR
KH_2_PO_4_	0.37	Sigma Aldrich
MgSO_4_	0.2	Sigma Aldrich
NaHCO_3_	25	VWR
Na_2_-EDTA	0.014	Sigma Aldrich
C_5_H_10_N_2_O_3_	1	Gibco, Thermo Fisher scientific, Waltham, Massachusetts, USA
NaC_3_H_5_O_3_	1.67	Sigma Aldrich
C_3_H_3_NaO_3_	0.18	Gibco
C_6_H_12_O_6_	0.2	Sigma Aldrich

### Statistical analysis

All statistical tests were done using JMP Software (v.14.0.0, SAS Institute Inc.). Multiple comparisons have been done using a Dunnett’s multiple comparison test when comparisons are performed toward a control condition. Otherwise, analyzes were performed using a Welch t-test, an O’Brien or a Wilcoxon test. We have indicated in the text the appropriate test used. The threshold for statistical significance is set to a p value ≤0.05.

## Results

### ARMC2 is expressed in mouse ciliated tissues

**FIGURE 1:**
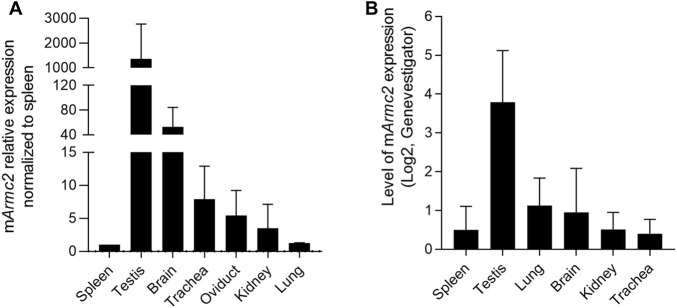
*Armc2* is expressed in testis and ciliated tissues. **(A)** Mouse *Armc2* expression in kidney, testis, oviduct, brain, lung and trachea relative to its expression in spleen. Expression was quantified by RT-qPCR normalized regarding the *Atcb* gene as a reference control. Measurements were performed 3 times. **(B)** Levels of *Armc2* expression in mouse tissues obtained by Affymetrix studies and recovered from Genevestigator TM (https://genevestigator.com/).

We assessed *Armc2* expression across multiple adult mouse tissues by RT-qPCR. As expected, expression was highest in testis, where *Armc2* mRNA level was >25-fold higher than in brain, the next most highly expressing tissue (Figure 1A). Elevated expression was also found in tissues harboring multiciliated cells (trachea > oviduct > lung), as well as in tissues with primary cilia (kidney > spleen). Our results are in accordance with released transcriptomic datasets (Genevestigator, Figure 1B).

RT-qPCR demonstrates that ARMC2 is expressed in ciliated tissues, prompting us to investigate its roles in multiciliated epithelia of the upper respiratory tract, the oviduct, and the brain ependyma.

### The *lack of* ARMC2 impairs tracheal ciliogenesis and ciliary function

**FIGURE 2:**
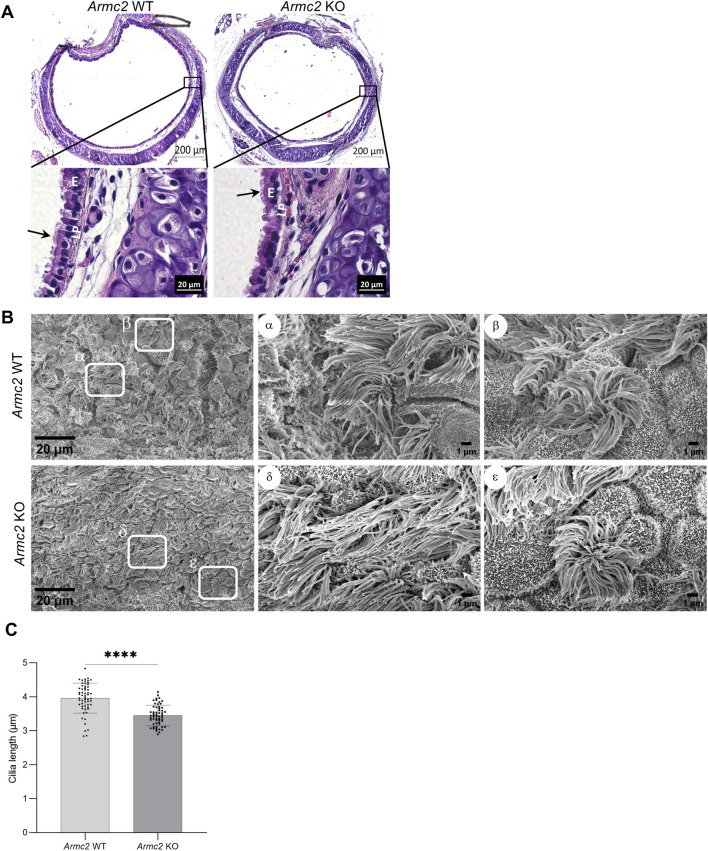
The *Armc2* deletion influences the tracheal cilia formation. **(A)** Histological analysis of tracheal tissues (hematoxylin-eosin staining) reveals no apparent defects in the *Armc2*-KO mice. The brush borders are observable in enlarged images and cilia are indicated by the arrows. E: epithelium, LP: Lamina propria. **(B)** Scanning electron microscopy analyses of the tracheal cilia for both *Armc2*-WT and *Armc2*-KO mice reveal no defect in the density of the MCCs or in the cilia orientation. Enlargements for both Armc2-WT (a, b) and *Armc2*-KO (d, e) mice show normal cilia morphology. **(C)** Quantification of the tracheal cilia length shows that cilia are shorter in *Armc2*-KO mice (dark grey) than in *Armc2*-WT (light grey) mice (****p < 0.0001, Welch t-test n = 177 for the *Armc2*-WT and n = 165 for the *Armc2*-KO mice, from 4 independent mice in each genotype).

To assess the impact of ARMC2 loss on motile ciliogenesis, we used our previously published *Armc2* MMAF mice model (Coutton et al., 2019). We first performed a comparative histological analysis of tracheas from *Armc2*-WT (wild type) and *-*KO (knock out) animals. Representative images are provided in Figure 2. Transverse sections revealed uniform tunica mucosa comprising the airway epithelium (E, Figure 2A) and lamina propria (LP, Figure 2A) facing the lumen, in both *Armc2*-WT and -KO mice (Figure 2A). The cilia and the ciliated pseudostratified columnar epithelia (arrows) were well defined in both WT and KO tracheas (Figure 2A, lower images). Thus, no overt abnormalities were detected at the histological level.

To examine ciliary ultrastructure at higher resolution, we next performed scanning electron microscopy (SEM). No obvious differences were observed in the number of ciliated cells present in the mucosa (Figure 2B). Cilia were uniformly oriented in the same direction (Figure 2B α and δ) and morphologically well formed (Figure 2B β and ε). However, quantitative measurements revealed a significant reduction of 12% in ciliary length in *Armc2*-KO mice: mean length was 3.46 ± 0.50 μm in KO versus 4.02 ± 0.64 μm in the *Armc2*-WT mice (Figure 2C).

**FIGURE 3:**
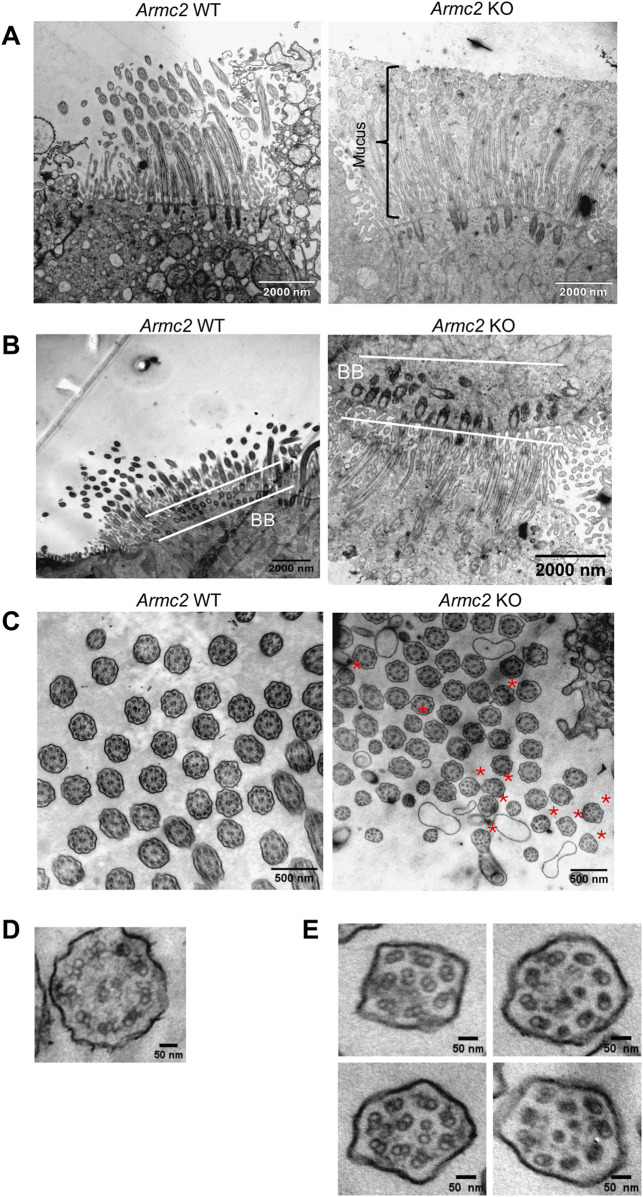
The *Armc2* deletion influences the tracheal cilia structure. **(A)** Transmission electron microscopy (TEM) analysis shows some mucus accumulation in the trachea of *Armc2*-KO mice. **(B)** TEM analysis showing the basal bodies (BB) disorganization in the *Armc2*-KO mice. **(C)**, **(D)** and **(E)** TEM analysis of the tracheal axonemes showing CPA defaults and symmetry loss in the *Armc2*-KO animals. Normal axoneme is shown in **(D)**. Abnormal axonemes indicated by a red asterisk are zoomed in **(E)**.

Cilia ultrastructure was next investigated by transmission electron microscopy (TEM) (Figure 3). Evidence of impaired mucociliary clearance was observed in *Armc2*-KO tracheas: in eight of 105 KO mice (7.6%), cilia were embedded in mucus (visible as electron-dense/granular material surrounding the axonemes), a feature never detected in WT animals (0 of 121) (Figure 3A). These observations indicate that loss of ARMC2 has measurable functional consequences for tracheal ciliary activity. We also observed an altered distribution of docked basal bodies. In a subset of KO samples, the basal-body implantation zone was expanded (delineated by white lines), consistent with disturbed basal-body positioning; misaligned basal bodies were observed in four of 105 (3.6%) *Armc2*-KO animals, compared with only one of 121 (0.8%) WT animals (Figure 3B). Consistent with these defects, transversal sections evidenced abnormal axonemal structures (Figures 3C,D) in *Armc2*-KO cilia, including severe disruption of the canonical 9 + 2 structure (Figure 3E), loss of the central pair with associated symmetry defects (Figure 3E). Quantification showed a ∼30-fold increase in abnormal axonemes in KO tracheas (9.85% ± 1.95%, mean ± SD, *n* = 3 mice) compared to the WT (0.33% ± 0.3%, mean ± SD, *n* = 3 mice).

**FIGURE 4:**
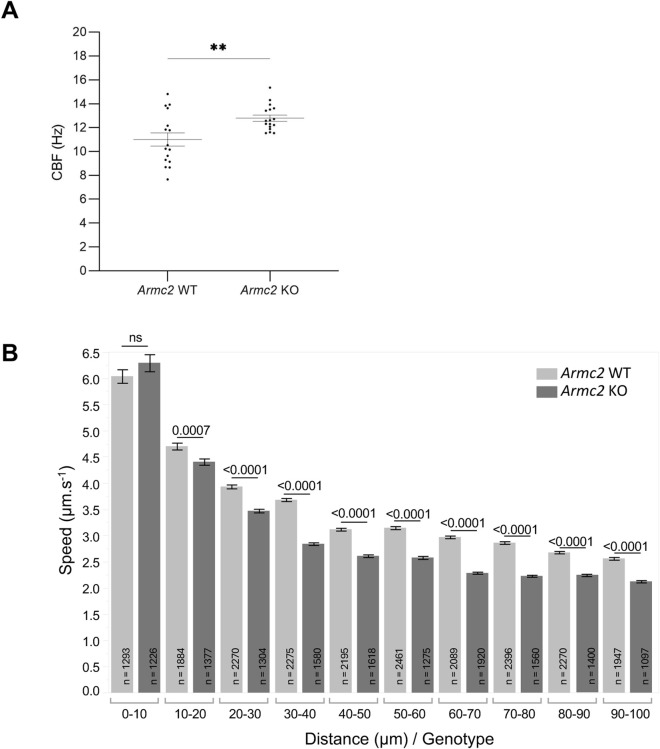
The *Armc2* deletion influences the tracheal cilia function. **(A)** Tracheal cilia in Armc2-KO mice beat with increased frequency. (**p = 0.0112, Welch t-test, n = 72 for the *Armc2*-WT from seven independent mice, and n = 56 for the *Armc2*-KO from four independent mice). **(B)** The *Armc2*-KO (dark grey) tracheal cilia are less effective in term of flow velocity. Results for *Armc2*-WT are represented in light grey and for *Armc2*-KO mice in dark grey. Numbers of analyzed beads are indicated for each genotype and distance. 16,040 beads’ tracks were analyzed for the *Armc2*-WT and 15,429 for *Armc2*-KO mice, five mice for each genotype. Welch t-test, p value as indicated, ns = not significant.

We next assessed consequences of ARMC2 loss on ciliary beating and flow generation under conditions mimicking physiological viscosity by supplementing the medium with 0.5% methylcellulose. Tracheal ciliary beat frequency (CBF) differed between genotypes, with mean values of 10.93 ± 4.56 Hz in WT mice and 12.68 ± 3.15 Hz in KO mice (Figure 4A). To directly test cilia’s ability to generate fluid flow, we tracked bead displacement and quantified bead velocity as a function of distance from the ciliated edge (Figure 4B). At the immediate ciliary edge, flow speed was maximal and did not differ significantly between genotypes (6.03 ± 4.64 μm/s in WT versus 6.29 ± 5.70 μm/s in KO). In contrast, at distances of 10–100 μm from the ciliary edge, flow velocities were consistently and significantly reduced in *Armc2*-KO explants compared with WT. These data indicate that ARMC2 is required for tracheal cilia to generate robust, long-range optimal fluid flow, despite preserved near-field flow at the ciliary border.

Altogether, these data indicate that *Armc2* contributes to tracheal cilia formation and is essential for maintaining normal tracheal ciliary beating and generating an optimal flow speed, and consequently efficient mucociliary clearance.

### *Armc2*-KO females are sub-fertile

**FIGURE 5:**
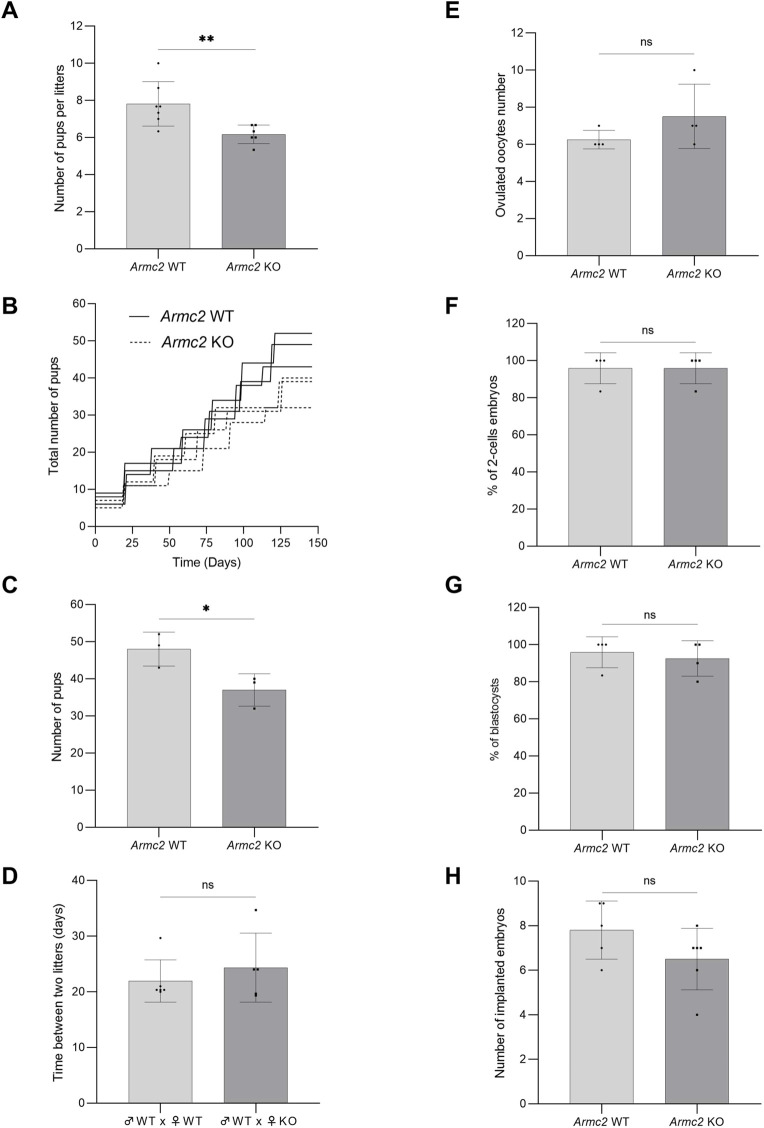
The *Armc2*-KO females are sub-fertile. **(A)** The number of pups per litter is reduced in the *Armc2*-KO females (**p = 0.009, Welch t-test, n = 19 litters for 3 *Armc2*-WT females and n = 18 litters for 3 *Armc2*-KO females). **(B)** Comparative accumulation of live pups recorded over a period of 130 days for both *Armc2*-WT and *Armc2*-KO mice crossed with WT males shows sub-fertility of *Armc2*-KO females. **(C)** The total number of pups is reduced in the *Armc2*-KO females (p = 0.0396, Welch t-test, from three independent mice in each genotype). **(D)** The time between 2 litters is identical for both *Armc2*-WT and *Armc2*-KO mice (p = 0.3463, Welch t-test, from three independent mice in each genotype). **(E)** Numbers of collected eggs in *Armc2*-WT and *Armc2*-KO females showed that the *Armc2* deletion has no effect on oocyte production (p = 1, Welch t-test, from four independent mice in each genotype). **(F)** The % of 2-cells embryos regarding the ovulated eggs showed that almost all oocytes were fertilized upon natural mating with WT males (p = 0.2474, welch t-test, from four independent mice in each genotype). **(G)** The % of blastocysts regarding the 2-cell embryo number demonstrated that almost all the 2-cell develop until the blastocyst stage (p = 0.6186, Welch t-test, from four independent mice in each genotype). **(H)** The embryo implantation rate is not significantly reduced in the *Armc2*-KO females (p = 0.1439, Welch t-test, from 5 *Armc2*-WT and 6 *Armc2*-KO mice). For each graphical representation, results for *Armc2*-WT are represented in light grey and for *Armc2*-KO mice in dark grey.

**FIGURE 6:**
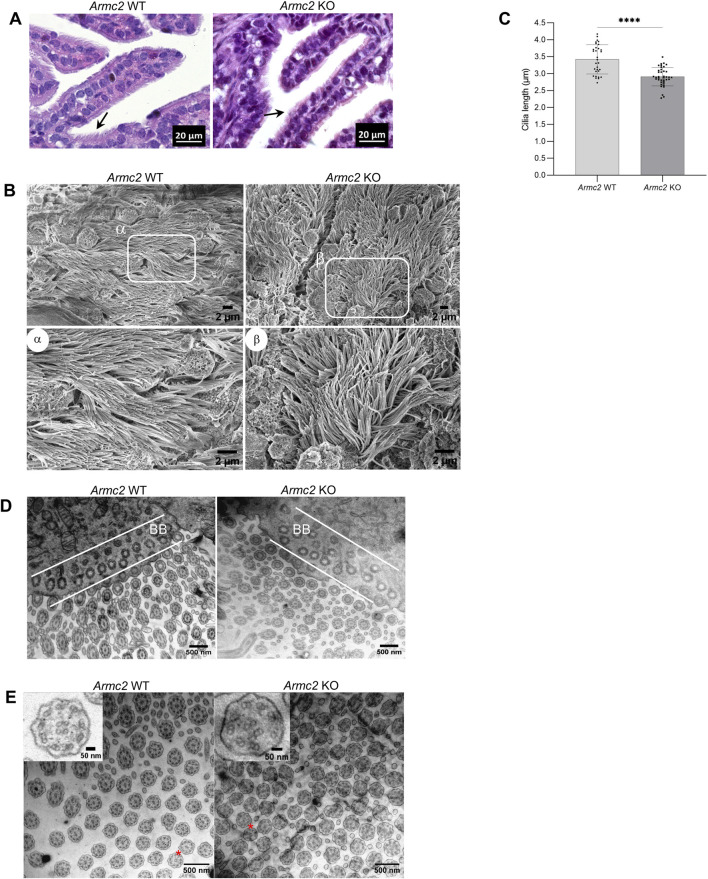
Oviductal cilia length is impacted in *Armc2*-KO females. **(A)** Histological analysis of the oviductal tissue shows no apparent defect in the *Armc2*-KO animals (hematoxylin-eosin staining). Arrows indicate the cilia border. **(B)** SEM analysis of the tracheal cilia for both *Armc2*-WT and *Armc2*-KO mice reveals no defect in the density of the MCCs or in the cilia orientation. Enlargements α and β are for *Armc2*-WT and *Armc2*-KO, respectively. **(C)** *Armc2* deletion decreases oviductal cilia length. Data for *Armc2*-WT are represented in light grey and for *Armc2*-KO in dark grey (****p < 0.0001, Welch t-test, n = 73 from three independent *Armc2*-WT mice and n = 117 from four independent *Armc2*-KO mice) **(D)** TEM analysis reveals no disorganization of the basal bodies (BB) in the *Armc2*- KO animals. **(E)** TEM analysis of the oviductal axonemes shows no defect in the *Armc2*-KO animals. Abnormal axonemes highlighted by a red asterisk are enlarged in the insets.

Motile cilia are not only present in upper-airways tissues but also in the female reproductive tract. In our initial characterization of the *Armc2*-KO line, we observed a modest reduction in female fertility (Coutton et al., 2019). We therefore reassessed in detail the female mice reproductive phenotype by crossing *Armc2*-WT or *Armc2*-KO females with WT males. Consistent with our previous report, *Armc2*-KO females produced fewer pups per litter (6.17 ± 1.38) than *Armc2*-WT females (7.58 ± 1.71) (Figure 5A). Over the 130-day monitoring period, the total number pups produced by Armc2-KO mice was significantly reduced (with a mean of 37 ± 4.36) compared to *Armc2*-WT mice (48 ± 4.58 pups) (Figures 5B, 6C). This decrease rather cannot be attributable to a reduction in the number of litters, which was similar in both groups (mean 6 litters; data not shown). The inter-litter interval showed a modest, non-significant increase in KO females (24.33 ± 7.33 days) compared to WT (22.06 ± 5.68 days) (Figure 5D). We then looked for the number of ovulated eggs and their transport into the oviduct. Females were paired with WT males overnight, and upon plug detection the following morning, eggs/zygotes were recovered from the ampulla and counted (n = 4 females per genotype). The number of recovered eggs/zygotes was not significantly different (6.25 ± 0.50 in WT *versus* 7.50 ± 1.73 in KO) (Figure 5E). Fertilization rates were comparable, with ∼90% of eggs fertilized in both genotypes (Figure 5F), and most zygotes progressed to the blastocyst stage (Figure 5G), indicating no overt defect in fertilization or preimplantation development under these conditions. Finally, implantation was assessed at embryonic day 6; the number of implanted embryos was slightly lower in KO females (6.5 ± 1.37, n = 5) than in WT (7.8 ± 1.3, n = 6), but this difference was not statistically significant (Figure 5H).

**FIGURE 7:**
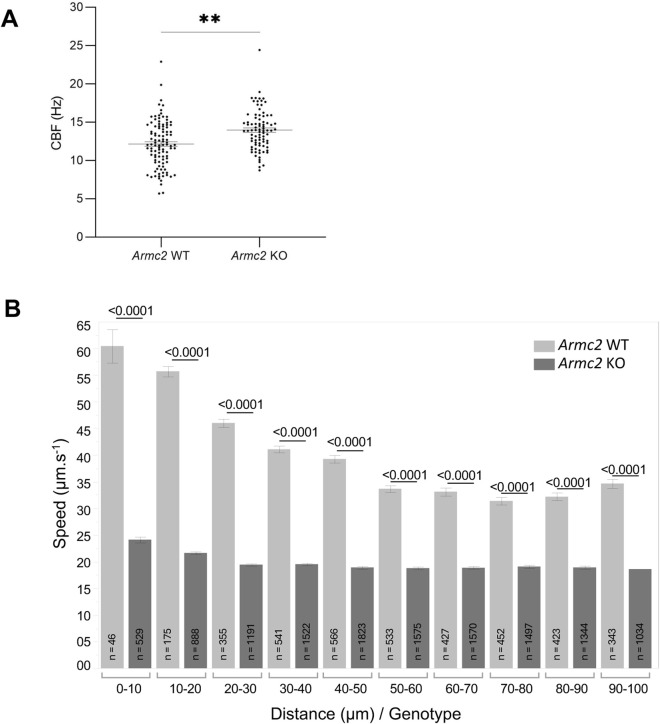
The *Armc2* deletion influences the oviductal cilia function. **(A)** Oviductal ciliary beat frequency is increased in *Armc2-*KO females. Data for *Armc2*-WT are represented in light grey and for *Armc2*-KO in dark grey (**p = 0.0195, Welch t-test n = 276 from four independent *Armc2*-WT mice and n = 276 from four independent *Armc2*-KO mice). **(B)** The *Armc2*-KO (dark grey) oviductal cilia are less effective in term of flow velocity. Results for *Armc2*-WT are represented in light grey and for *Armc2*-KO mice in dark grey. Numbers of analyzed beads are indicated for each genotype and distance. 4354 beads’ tracks were analyzed for the *Armc2*-WT and 14,669 for *Armc2*-KO mice, four mice for each genotype. Welch t-test, p value as indicated, ns = not significant.

To pinpoint the basis of the observed female subfertility, we examined the oviduct epithelium. Histological analysis of the ampulla revealed no overt differences between *Armc2*-KO and -WT females (Figure 6A). Likewise, oviductal cilia appeared morphologically intact and well formed in the absence of ARMC2 (Figure 6B). Quantitative measurements nonetheless showed that cilia were significantly shorter in *Armc2*-KO mice (2.97 ± 0.42 µm) compared with WT (3.37 ± 0.60 µm) (Figure 6C). In contrast to the trachea, ultrastructural analysis revealed no abnormalities in the alignment of basal bodies (Figure 6D), and no axonemal defects (less than 0.5% for both genotypes, Figure 6E). Despite this preserved ultrastructure, ciliary function was altered. The CBF differed significantly between the *Armc2*-WT (mean of 12.62 ± 5.00 Hz) and *Armc2*-KO (mean of 13.54 ± 4.21 Hz) animals (Figure 7A). Moreover, bead-tracking assays showed that flow velocity was consistently reduced in *Armc2*-KO oviducts, including at the cilia border: within 0–10 μm, mean velocity was 24.11 ± 12.09 μm/s in KO versus 60.38 ± 21.30 μm/s in WT (Figure 7B). However, these differences do not impact the eggs collection (Figure 5E).

Together, these data indicate that ARMC2 loss leads to subtle ciliogenesis defects (reduced cilia length) and measurable functional impairment of oviductal flow, which likely contributes to female subfertility. Unlike the upper airway, however, oviductal cilia do not display overt basal body or axonemal structural abnormalities. The trend toward reduced implantation could reflect compromised cilia-dependent transport-potentially delaying embryo transit to the uterus and thereby reducing synchronization with the optimal implantation window.

### *Armc2*-KO mice harbor *situs ambiguus* and hydrocephalus

**FIGURE 8:**
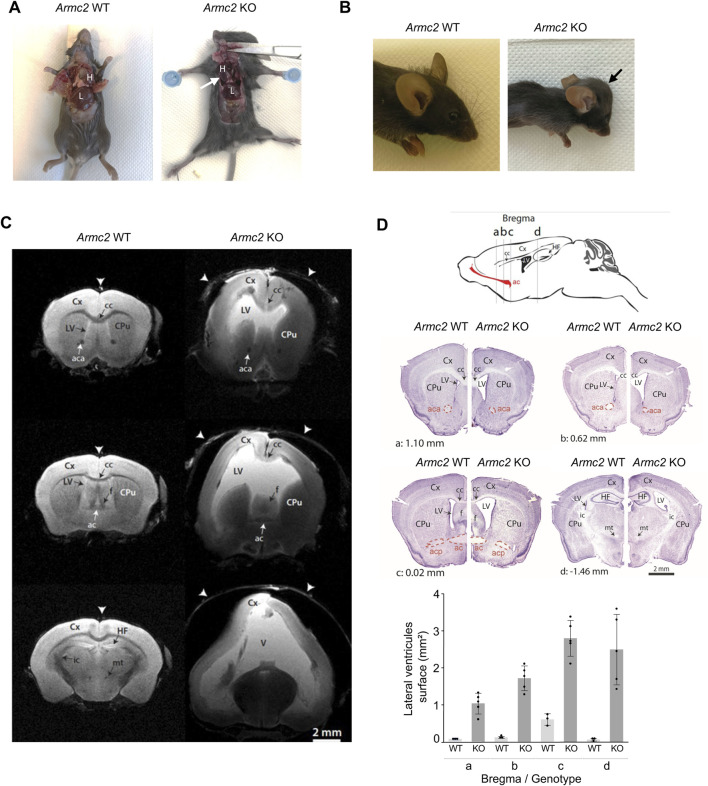
The *Armc2-KO* mice present *situs ambiguus,* ventricular enlargement and hydrocephalus **(A)** *Situs ambiguus* is detected in the *Armc2*-KO mice (white arrow). H: heart, L: liver **(B)** Hydrocephalus is detected in the *Armc2*-KO mice (black arrow). **(C)** Sagittal diagram of mouse brain indicating the position relative to the Bregma of the four slices **(A-D)** as well as the localization of the anterior commissures represented in red. Cresyl violet-stained coronal half-sections of *Armc2*-WT and *Armc2*-KO mouse brains in planes a, b, c, and d as indicated in the upper sagittal diagram. The anterior commissures (ac, aca and acp) are delineated by dashed red lines. The graph represents mean lateral ventricular area in the four planes. In all section planes, the lateral ventricles (LV) are consistently enlarged in the *Armc2*-KO mice (dark grey) compared to the *Armc2*-WT mice (light grey). a: p = 0.0014; b: p = 0.0004; c: p =0.0002; d: p = 0.0046; Welch t-tests from three independent *Armc2*-WT mice and from five independent *Armc2*-KO mice. **(D)** T2-weighted magnetic resonance images show significant enlargement of the cerebral ventricles associated with skull deformation (see arrowheads) in an *Armc2*-KO mouse with severe hydrocephalus. Abbreviations: ac, anterior commissures; aca, anterior part of anterior commissures; acp, posterior part of anterior commissure; cc, corpus callosum; CPu, caudate putamen; Cx, cortex; f, fornix; HF, hippocampal formation; ic, internal capsule; LV, lateral ventricle; mt, mammillary tract.

In addition to chronic upper and lower airway disease such as chronic sinusitis and bronchiectasis, PCD is also associated with less frequent manifestations such as situs inversus, where internal organs are in opposite positions of what is normal, and hydrocephalus. Interestingly, during colony monitoring, we observed organ laterality defects with situs ambiguus, with dextroposition of the heart (right-sided orientation) in 0.7% of the *Armc2*-KO animals (2/268) whereas this defect was never observed in control animals (Figure 8A). Hydrocephalus was also noticed, characterized by a dramatic size increase of the cranial vault, in 1.5% (4/268) of the *Armc2*-KO, 0.6% (3/483) of the *Armc2*-heterozygous mice (Figure 8B) and 0% of the WT animals (n = 107). MRI analysis of a KO mouse with overt hydrocephalus, revealed pronounced cerebrospinal fluid accumulation accompanied by cranial deformation (Figure 8C). To better characterize the observed hydrocephalus, we measured ventricular dimensions on brain coronal sections in *Armc2*-WT and -KO mice of 3 months of age that did not show overt hydrocephalus. Strikingly, all *Armc2*-KO mice examined exhibited ventricular dilation in comparison to *Armc2*-WT (Figure 8D; Table 4). Together, these findings indicate that ARMC2 is required for proper function of nodal cilia (left–right patterning) and ependymal motile cilia (CSF circulation/clearance), extending its role beyond the reproductive tract and airways.

**TABLE 4:** Brain ventricular dimensions in *Armc2*-WT and -KO mice regarding coronal sections.

Coronal section	Armc2-WT	Armc2-KO
a	0.08 ± 0.03	3.00 ± 0.28
b	0.12 ± 0.02	1.72 ± 0.33
c	0.60 ± 0.16	2.80 ± 0.49
d	0.06 ± 0.03	2.49 ± 0.95

### RSPH1 expression is altered in tracheal ciliated cells from *Armc2*-KO animals and *in silico* analyses predict ARMC2 and RSPH1 interaction

**FIGURE 9:**
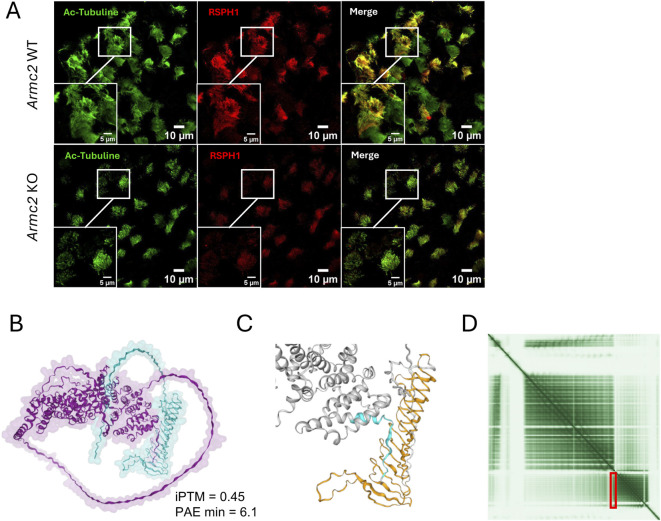
*ARMC2* and RSPH1 functional and molecular interactions in mammalian trachea. **(A)** RSPH1 is less abundant in the tracheal cilia of *Armc2*-knockout animals. Immunofluorescence staining of tracheal explants reveals the ciliary border, as highlighted by acetylated tubulin (green), and the localization of RSPH1 (red). **(B)** Predicted structural model of the complex (iPTM = 0.45), showing ARMC2 (purple) and RSPH1 (cyan). **(C)** Close-up view of the local interface patch involving the last amino acids of Armc2 (C-terminus, blue) and the first amino acids of Rsph1 (N-terminus, orange). **(D)** PAE matrix consistent with a transient interaction (PAE min = 6.1).

In *Chlamydomonas*, ARMC2 functions as a transport adaptor for RS precursors (Lechtreck et al., 2022). To investigate if the function of ARMC2 is conserved in mouse we examined the expression of RSPH1 and RSPH4 in tracheal explants from *Armc2*-WT or -KO animals using immunofluorescence. We never succeed to detect ARMC2 in trachea, or oviduct extracts by IF and Western blot with commercially available anti-ARMC2 antibodies. This is likely due to the strong reduction of ARMC2 expression in mature ciliated cells. A reduction in RSPH1 expression was detected in *Armc2*-KO animals (Figure 9A), prompting us to investigate their potential interaction more thoroughly. A predicted structural model (Figure 9B) suggests that ARMC2 and RSPH1 interact (iPTM = 0.45), with the C-terminus of ARMC2 binding to the N-terminus of RSPH1 (Figure 9C). Evidence of a transient interaction (PAE min = 6.1) is shown in Figure 3D. In contrast, no difference was observed in RSPH4A expression (Supplementary Figure 1).

## Discussion

MMAF syndrome is characterized by heterogeneous structural defects of the sperm flagellum and a major cause of male infertility. Because motile cilia and flagella share the axoneme structural core with its canonical microtubular 9 + 2 organization, MMAF and PCD can sometimes overlap. Indeed, among the 50 genes currently linked to MMAF, at least 16 have also been implicated in PCD. Conversely, variants in established PCD genes can be associated with male infertility, although sperm morphology is often incompletely characterized in these reports.

We have previously demonstrated that ARMC2 is a causal MMAF gene in humans and that *Armc2* is essential for sperm flagellum formation in mice: *Armc2-*KO males display a typical MMAF phenotype and are infertile (Coutton et al., 2019). The aim of the present study was therefore to determine whether the MMAF-IFT *Armc2* gene also contributes to ciliogenesis and by extension to PCD-like phenotypes.

### Impaired cilia structure and function in upper airways from *Armc2*-KO animals

We conducted histological, morphological and functional analysis of trachea motile cilia from WT and *Armc2*-KO animals. At the structural level, we observed basal body misalignment. Because basal bodies derive from centrioles (Breslow and Holland, 2019), these observations raise the possibility that ARMC2 contributes to efficient centriole docking and/or positioning at the apical membrane during basal body formation. Comparable basal body misalignment have been reported upon perturbations of other basal body-associated proteins such as *Cby* (Yang et al., 2014; Siller et al., 2015) and also in mutants of ciliary kinesins such as *kif3a*, which, beyond its role in IFT, has been involved in basal body anchoring in zebrafish photoreceptors (Pooranachandran and Malicki, 2016). Within the axoneme of tracheal cilia, we detected microtubules disorganization and symmetry loss. Similar microtubules defects have been described in mouse models with impaired IFT (Yang et al., 2014), but they are not unique to IFT disruption, as related ultrastructural abnormalities have also been reported in mutants affecting multiciliated cell differentiation such as *Ulk4* (Liu et al., 2016), or the nexin-dynein regulatory complex (N-DRC) such as *Gas8* (Lewis et al., 2016). We also observed central pair apparatus (CPA) defects, similarly to the CPA loss previously reported in *Armc2*-KO sperm flagella (Coutton et al., 2019). CPA abnormalities are frequently observed in mice lacking CPA components like *Spag6* (Teves et al., 2014) or *Spag17* (Teves et al., 2013) and in mutants affecting radial spoke (RS) proteins such as *RSPH1* (Kott et al., 2013; Yin et al., 2019). Notably, the CPA phenotype observed upon ARMC2 loss may relate to the *Armc2* function described in *Chlamydomonas* as a cargo adapter for IFT-mediated transport of RS (Lechtreck et al., 2022). Such defects could contribute to altered beating dynamics, as loss of the CPA or RS is known to shift motile cilia from planar to more helicoidal waveforms (Lindemann and Lesich, 2024).

We also found that tracheal cilia were significantly shorter in *Armc2*-KO mice. This is in accordance with the function of the IFT genes and has been reported for RPE-1 cells upon silencing of IFT-*Cyld* gene (Yang et al., 2014) and in mouse ependymal cells after depletion of the multiciliated-cell differentiation factor *Rfx3* (El Zein et al., 2009). Reduced ciliary length may also contribute to the impaired mucociliary clearance we observed (Rautiainen et al., 1991; Smith et al., 2008). Indeed, we detected a 12% reduction in ciliary length in *Armc2*-KO animals compared with wild-type controls. This magnitude is comparable to the differences reported between smokers and non-smokers, approximately 15% in endobronchial biopsies and 9% in isolated cilia, differences for which existing models predict a measurable decline in mucociliary clearance (Leopold et al., 2009). Additionally, the observed 0.56 µm difference is comparable to the length difference seen in the *Prom1*-KO model (0.6 µm). In that case, the reduced cilia length impaired ciliary beat frequency (CBF) and mucociliary clearance (Serra et al., 2022). Broadly, this suggests that any disruption in cilia length-whether an increase or a decrease-negatively impacts mucus clearance.

To quantify ciliary motility under more physiologically relevant mechanical load, we measured CBF in medium containing 0.5% methylcellulose to increase viscosity. This is particularly relevant in the context of PCD, which is associated with chronic airway inflammation and altered mucus properties (Legendre et al., 2021). Under these conditions, CBF was significantly higher in *Armc2*-KO than in *Armc2*-WT mice. This finding was unexpected, as previous study on others IFT-deleted gene mouse models, such as *Ift88* (Gilley et al., 2014) reported decreased tracheal and ependymal CBF, respectively. However, an elevated CBF in the setting of shortened cilia is consistent with establish observations that shorter, immature cilia tend to beat faster than longer, fully mature cilia during *in vitro* ciliogenesis (Oltean et al., 2018).

Here, the *Armc2* deletion impacts the ability of tracheal cilia to generate an effective power stroke. Although beating persists, the propulsive force (captured by the measured bead/flow velocity) was significantly reduced in *Armc2*-KO explants, providing mechanistic basis for the mucus accumulation observed. This is especially relevant given that the airway surface liquid/mucus layer (epiphase) in rodents can vary widely, from ∼0.1 to 50 µm (Sims and Horne, 1997).

Overall, the reduced flow velocity in the *Armc2*-KO mice correlates with the observed diminished cilia length observed as found for *C. reinhardtii* (Bottier et al., 2019) or for the immature, consequently shorter, tracheal cilia of mice (Francis et al., 2009).

Collectively, these findings highlight the essential role of ARMC2 in tracheal ciliogenesis and in sustaining the effective motility required for robust mucociliary clearance.

### Despite female subfertility, oviductal cilia structure appears preserved in *Armc2*-KO animals

Given that dysfunction of oviductal MCCs can compromise female infertility, we conducted an extended breeding analysis and confirmed that *Armc2-*KO females are subfertile, producing fewer pups over time. Female subfertility is also well documented in PCD: pregnancy is reported in 39% of women suffering from PCD during their first year of attempting to conceive compared to the general population (>70%) (Vanaken et al., 2017). More broadly, a recent synthesis of the literature concludes that subfertility is common in PCD and likely reflects impaired motility of reproductive-tract cilia and/or sperm flagella, with substantial variability across genotypes and studies (Newman et al., 2023). The same affected gene can be associated with male and female sub-fertilities such as *RSPH1* (Onoufriadis et al., 2014) or *DNAH11* (Schwabe et al., 2008; Vanaken et al., 2017). Genes can also be involved only in female subfertility such as *CCDC151* (Wang et al., 2020) or only in male infertility such as *GAS5* (Vanaken et al., 2017). More genes have been reported for men sub-fertilities (28 genes) than for women sub-fertilities (18 genes) (Newman et al., 2023). In this context, our findings argue that ARMC2 should be considered not only an MMAF gene relevant to male infertility, but also a candidate determinant of female subfertility, consistent with its role in motile cilia-dependent physiology.

To dissect the basis of the female subfertility, we conducted the same morphological and functional analysis as for the trachea. Oviductal cilia shared two key phenotypes observed in the airways: reduced ciliary length and increased CBF (measured in the infundibulum). However, in contrast to the upper-airway cilia, we did not detect apparent defects in basal body alignment or axonemal ultrastructure. We then sought to identify which step of reproduction might be affected. The subfertility phenotype was not attributable to impaired eggs collection, indicating that the flow generated at the infundibulum was sufficient to collect cumulus-oocyte complexes in *Armc2*-KO females. Likewise, we did not observed evidence for impaired embryo development. By contrast, we have observed a trend toward a reduction in the number of embryos implanted, suggesting a defect downstream of fertilization and early cleavage. This raises the possibility of compromised function of the posterior oviduct, where cilia-driven flow contributes to embryo transport toward the uterus (Niwa et al., 2012). Consistent with the importance of oviductal cilia for reproductive output, the number of pups is reduced in the miR-34b/c and miR-449a/b/c double KO mouse model which lacks oviductal cilia (Yuan et al., 2021). Together, these findings support a model in which female subfertility in *Armc2*-KO females results from functional impairment of oviductal cilia, particularly in posterior region, leading to delayed embryo transit. Such defects could delay embryo arrival into the uterus outside the optimal implantation window, thereby lowering implantation rates.

### *Armc2* is a PCD-associated gene

Beyond airway and reproductive phenotypes, we observed additional hallmarks of motile ciliopathies during colony monitoring, including situs ambiguus and hydrocephalus, features classically linked to impaired nodal and ependymal cilia, respectively (Hjeij et al., 2013). In our cohort, situs ambiguus (dextroposition of the heart) was detected in 0.7% of *Armc2*-KO animals (2/268) and was not observed in controls. Overt hydrocephalus occurred in 1.5% of KO mice (4/268) and 0.6% of heterozygotes (3/483), but not in WT animals (0/107). Importantly, this prevalence likely underestimates the true penetrance, as only clinically apparent cases were recorded; consistent with this, histological analyses of KO brains without apparent cranial enlargement revealed ventricular dilation in all animals examined (5/5).

Hydrocephalus and laterality defects are recurrent outcomes in mouse models with disrupted motile-cilia biogenesis or function. For example, multiciliated-cell–specific CEP164 loss recapitulates PCD-like features including hydrocephalus and infertility, while mutations affecting Wdr69/DAW1 are linked to laterality defects (including heterotaxy) and abnormal ciliary motility (Lee and Ostrowski, 2021). Defective ependymal cilia function leading to communicating hydrocephalus has also been reported in NHERF1-deficient mice, and disruption of Jhy causes juvenile hydrocephalus associated with abnormal ciliary microtubule patterning and reduced motility (Lee and Ostrowski, 2021). Notably, hydrocephalus is described as sporadic in PCD patients but frequent in mouse motile-cilia models, underscoring species/model differences in penetrance and presentation. Given the consistent ventricular phenotype in *Armc2*-KO mice, it will be informative to assess neurobehavioral outcomes linked to disturbed CSF dynamics. Impaired brain clearance has been discussed as a contributor to accumulation of toxic metabolites, including amyloid-β (Tarasoff-Conway et al., 2015), in neurodegenerative contexts, and normal-pressure hydrocephalus is classically associated with a triad of gait disturbance, urinary incontinence, and cognitive impairment (Bonney et al., 2022). Collectively, our findings support *Armc2* as a gene whose loss can extend beyond MMAF to a broader motile ciliopathy/PCD-like spectrum, with implications for nodal and ependymal cilia-dependent processes. Thus, clinicians should be aware of the potential risk of hydrocephalus in a subset of MMAF patients harboring mutations in ciliogenesis genes.

### Absence of ARMC2 leads to decrease in RSPH1 expression in cilia trachea

Finally, we demonstrated that, albeit at much lower levels than in the testis, *Armc2* is expressed in tissues harboring multiciliated cells, including the trachea and the oviduct. Our modeling suggests possible interactions between ARMC2 and RSPH1, although further experimental studies, such as pull-down assays, are required to confirm these predictions. Together, these data strongly support that ARMC2 likely contributes to IFT in mammals, as previously demonstrated in *Chlamydomonas Algae* (Lechtreck et al., 2022).

### Novel Perspective on cilia Diversity and structural complexity

Our study highlights an underappreciated facet of ciliary biology: despite a shared core architecture and motility machinery, the phenotypic consequences of disrupting a single ciliary factor can be profoundly tissue specific. Loss of ARMC2 produces overt ultrastructural defects in tracheal multicilia, yet oviductal multicilia remain largely normal at the ultrastructural level despite clear functional impairment. This divergence underscores that ciliary assembly and motility are governed by context-dependent regulatory layers superimposed on conserved axonemal modules.

These findings support a model in which a broadly conserved molecular toolkit operates within organ-tailored constraints, including differences in ciliogenesis programs, mechanical load, luminal environment, and tissue-specific accessory structures. In this framework, ARMC2-dependent processes interface with local regulatory networks to shape ciliary output in a tissue-selective manner, providing an integrated view of how common ciliary components and their modulators collaborate dynamically to produce distinct physiological outcomes across organs.

## Conclusion

Our study demonstrates that the Armc2 is not only required for sperm flagellum assembly and function but also contributes broadly to the physiology of motile ciliated tissues. In sperm flagella and tracheal cilia, ARMC2 is required for maintenance of the CPA, a core axonemal structure essential for stability and efficient motility. In contrast, CPA architecture appears preserved in oviductal cilia despite clear functional deficit, suggesting the existence of tissue-specific compensatory or regulatory pathways in the female reproductive tract. The divergent outcomes across sperm, airway, and oviduct indicate that loss of *Armc2* results in context-dependent phenotypic outputs, reflecting organ-specific modulation of ciliary assembly and performance. In addition, our *in vivo* analyses support a critical role for ARMC2 in ependymal cilia-dependent cerebrospinal fluid homeostasis, consistent with the ventricular dilation and hydrocephalus phenotypes observed in mutant animals, a finding that may have broader implications for neurodevelopmental disorders and central nervous system homeostasis. The concomitant female subfertility further corroborates the involvement of *Armc2* in oviductal cilia functionality as evidenced by impaired ciliary beat frequency and disrupted fluid dynamics in the reproductive tract. Together, all these findings position *Armc2* as a determinant of PCD-like spectrum in mice. This newly uncovered role for ARMC2 implies that individuals diagnosed with MMAF due to variants in ciliogenesis-related genes may benefit from clinical awareness and, where appropriate, evaluation for PCD-associated complications, including neurological manifestations.

## Data Availability

The raw data supporting the conclusions of this article will be made available by the authors, without undue reservation.
