# Supplementary material for: ARMC2 loss impairs cilia structure and leads to primary ciliary dyskinesia symptoms in mouse organs
Source: Front Cell Dev Biol. 2026 May 26;14:1695239. doi: 10.3389/fcell.2026.1695239 (PMC13246696; doi:10.3389/fcell.2026.1695239)
Supplement: Supplementary file 1 [file Supplementaryfile1.pdf]

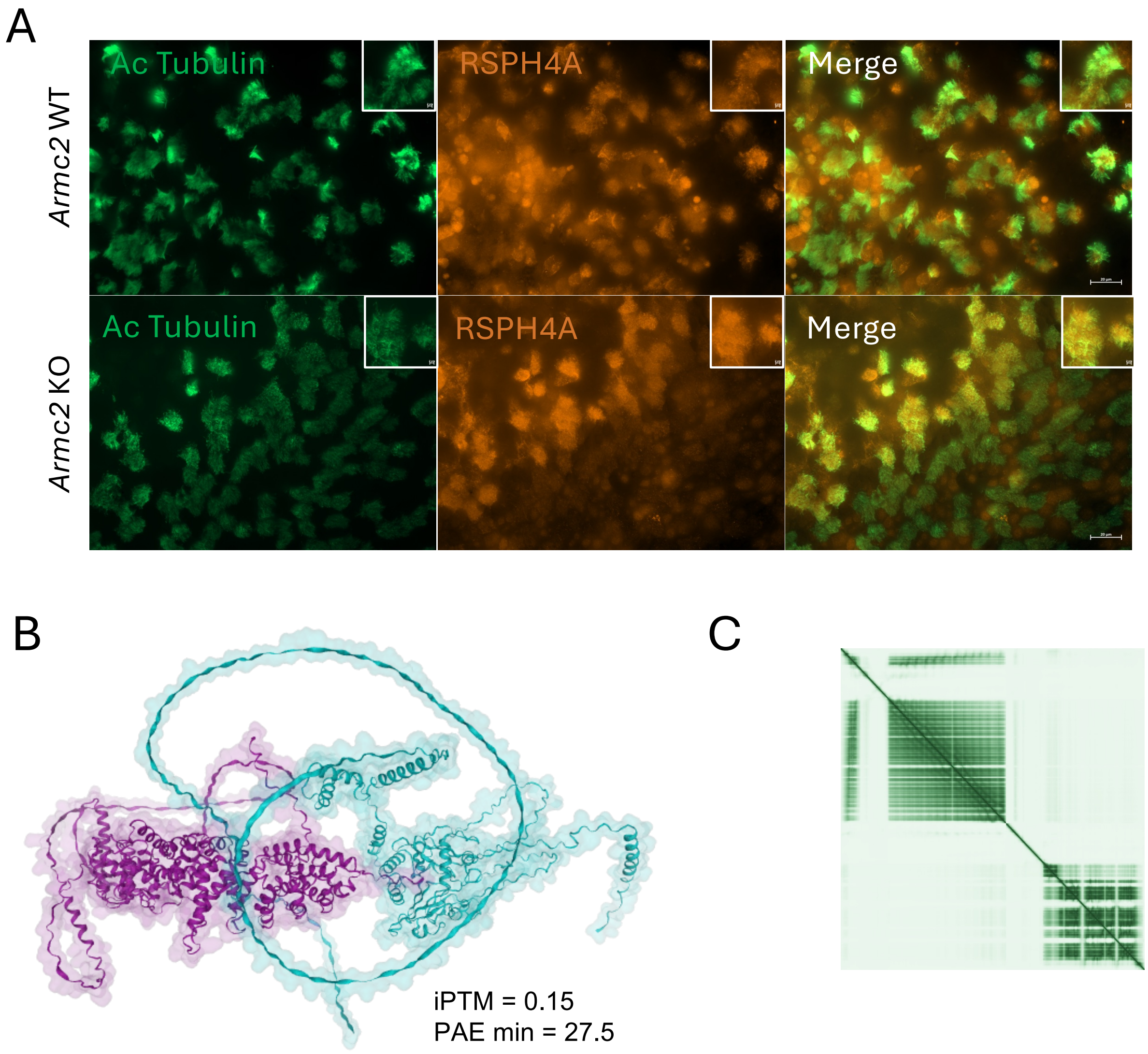

Supplemental Figure 1: Expression RSPH4 is not impacted by the absence of ARMC2 and no interaction was predicted by AlphaFold multimer analysis.
